# The rules of bereavement work: emotion work in online perinatal loss support groups

**DOI:** 10.1186/1471-2393-15-S1-A16

**Published:** 2015-04-15

**Authors:** Jessica Killeen

**Affiliations:** 1 Department of Law and Justice Studies, Avila University, Kansas City MO; Department of Sociology, Johnson County Community College, Overland Park KS, USA

## 

The success of pregnancy and infant loss support groups in helping parents, especially mothers, deal with the emotional trauma of stillbirth is well established in sociological and psychological research literature. More recent studies have focused on the emergence of virtual perinatal loss support groups and communities which offer a convenient means of accessing an extensive group of supporters who can help participants navigate self-care during bereavement, in addition to providing opportunities to engage in the collective grief work of traditional face-to-face groups [1,2].

Building on previous research into online perinatal support, the initial findings from an exploratory research study of mothers who have experienced the stillbirth of a child were reported, in particular the way in which bereaved mothers, both within and outside of online perinatal loss support groups, manage common emotional reactions to stillbirth. Mothers’ responses to emotions in themselves and in others which are identified as socially and personally problematic – guilt, shame and envy [3] were examined, in addition to the sociological concepts ‘feeling rules’ and ‘emotion work’ [4].

The usefulness of these concepts for a sociological understanding of guilt, shame and envy among bereaved mothers in online perinatal loss support groups was proposed. Just as therapists and scholars have identified the importance of grief work in the aftermath of a personal loss, sociologists have employed the concepts of ‘feeling rules’ and ‘emotion work’ to describe the way in which our culture dictates who may grieve, as well as where, when and how grief can occur. Stillbirth often involves changes to the social status and identity of the expectant mother, leading to ‘disenfranchised grief’. This is a situation in which emotions are inevitably in flux and support is most needed. However, the cultural restrictions or ‘feeling rules’ [4] around the open, public expression of mothers’ grief make the safety, privacy and validation of online support groups all the more necessary.

Furthermore, while the unwillingness of our culture to fully acknowledge the emotional trauma of stillbirth is well established in the research literature, it is not as well known how bereaved mothers apply these feeling rules to their own emotion work and to the emotional labor of other mothers, both within the online support community and outside of it. Examples drawn from a closed facebook perinatal support group of mothers who had experienced a stillbirth illustrate that there are clear feeling rules established within the group in relation to the emotions of guilt, shame and envy which are maintained by individuals and the group as a whole. Feeling rules are applied by the mothers to their own expressions of guilt, shame and envy, to other group members’ emotions and to the broader culture. Emotion work is undertaken on mothers’ own emotions as well as on the emotions of others in the group, which acts as a means of establishing trust within the group. As evidenced by excerpts from group members’ online exchanges, bereaved mothers comply with cultural feeling rules which suggest that stillbirth is a taboo subject while also desiring to challenge these rules.

As the research study is in the initial stages of data collection, the extent to which online support groups facilitate or challenge feeling rules around perinatal bereavement remains unclear. Preliminary data suggests that many bereaved mothers consider feelings of shame and envy unacceptable to share outside the support community, and these beliefs are reinforced by external cultural disapproval and internal validation within the group. However, it was concluded that further research is needed in this area.
